# Negligence, malpractice and recklessness committed by otorhinolaryngologists in the state of São Paulo-Brazil

**DOI:** 10.1016/j.bjorl.2026.101827

**Published:** 2026-05-22

**Authors:** Regina Maria Marquezini Chammes, Reginaldo Raimundo Fujita

**Affiliations:** a Universidade Federal de São Paulo (UNIFESP), São Paulo, SP, Brazil; b Conselho Regional de Medicina ‒ CREMESP management 2023‒2028, Brazil

**Keywords:** Medical errors, Otorrhynolaringology, Negligence, Malpratice, Imprudence, Recklessness

## Abstract

**Objective:**

The present study aims to survey the statistics of complaints of medical errors against otorhinolaryngologists in Regional Council of Medicine of the State of São Paulo (CREMESP), between the years 2010 and 2020.

**Methods:**

Inquiries and Lawsuits filed against otorhinolaryngologists working in the State of São Paulo, Brazil, between 2010 and 2020, at the CREMESP, manually, by the main plaintiff who is an elected Councilor and bound by confidentiality, were surveyed. The records were analyzed and separated according to the type of medical error reported: malpractice, recklessness or negligence and the subspecialties of otorhinolaryngology. The penalties applied to doctors found guilty at trial, with final and unappealable sentences, were also studied.

**Results:**

A total of 98 files were collected, of which 81 were archived and 17 were Professional Ethics Processes. Among the 81 inquiries involving medical error in one of the 3 modalities raised in the survey, 35 of them (43.2%) were complaints for negligence, 23 (28.3%) for malpractice and 23 (28.3%) for recklessness. The subspecialty with the most complaints for medical error was Otology and the ones that were most convicted were Rhinology and Pharyngology, making up 33% of the convictions each. The ethical penalties applied to doctors were mostly confidential penalties.

**Conclusion:**

The present study showed that Otorhinolaryngology is a specialty that presented an average of 8.9 complaints per year in the last decade (between 2010 and 2020) related to medical error, 82.6% of which were filed and 17.3% of them transformed into Professional Ethical Processes at CREMESP. Among the Professional Ethical Proceedings initiated, 52.9% were judged with a decision of guilt for the accused physician and 55.5% of these received culpability for violation of article 1° of the Code of Medical Ethics.

**Level of evidence:**

5.

## Highlights

• Negligence is the more frequent error committed by ENT in São Paulo State.
• Most of the ethical penalties applied to ENT for medical error, were confidential.
• Rhinology and Pharyngology were the areas with the highest number of convictions.
• Otology was the subspecialty with the highest number of complaints.

## Introduction

The “medical error” or “damage resulting from the provision of health services”, as it came to be called by the National Council of Justice (CNJ) in the classification system of lawsuits, is any medical conduct that deviates from scientific guidelines and good medical practices, resulting in harm to the patient, and can be classified into 3 types:

Negligence: It occurs when there is an omission of care, such as not performing necessary tests or ignoring relevant symptoms. It is a failure to properly monitor the patient. It is characterized by inaction, indolence, inertia, passivity. It is an omissive act. The abandonment of the patient, the omission of treatment.^1^

Recklessness: It is characterized by hasty action or lack of caution on the part of the health professional, putting the patient at risk.

Malpractice: It results from the lack of technical knowledge or ability to perform a certain procedure, especially in areas that require specialization.

According to the World Health Organization (2023), 1 in 10 patients suffer harm during medical care, and more than 3 million deaths occur annually due to unsafe care. In low- and middle-income countries, about 4 out of every 100 people die due to unsafe care.^2^^,^^3^

Patient harm potentially reduces global economic growth by 0.7% per year, with the global cost potentially reaching trillions of US dollars per year.^3^

Investing in reducing patient harm, in addition to being humanly and ethically correct, can generate significant financial savings to the health system in general, positively impacts health outcomes, reduces costs related to patient harm, improves the efficiency of the health system, and restores users' trust in the health service.^3^^,^^4^

Worl Health Organization (WHO) launched the Patient Safety Flagship as a transformative initiative to guide and support strategic actions for patient safety at the global levels, regional and national; with a primary focus on supporting the implementation of the Global Plan for Patient Safety 2021‒2030.^5^

In Brazil, for every 19.4 million people treated in hospitals each year, 1.3 million experiences at least one side effect caused by negligence or recklessness during medical treatment. In 2021, the Federal Council of Medicine reported that 92% of schools do not meet at least one of the 3 parameters considered ideal for adequate medical education. Every hour, three lawsuits for medical malpractice are distributed in Brazil. Implementing evidence-based medicine can reduce the number of medical errors and their consequences.^6^

With the decline in the quality of medical education in Brazil, attested by the Federal Council of Medicine, and the worrying exponential increase in the number of vacancies for medical courses in the country,^7^^,^^8^ there is concern about the quality of professionals who are being launched into the job market and the consequent increase in “medical error”.

Brazil recorded a significant increase in lawsuits related to medical errors in 2024, with a growth of 506% compared to the previous year, according to data from the National Council of Justice (CNJ), with 74,358 lawsuits in 2024, compared to 12,268 in 2023. In these lawsuits, moral and/or material damages are claimed. In the public health system, 10,881 lawsuits were filed for moral damages and 5854 for material damages. In the private system, these numbers are even higher, with 40,851 lawsuits for moral damages and 16,772 for material damages. 203 lawsuits were registered per day in 2024 in Brazil for medical error.^9^

It is important to emphasize that not every adverse or unwanted result can be truly characterized by poor professional practice. In order to characterize medical error, it is necessary to prove 3 factors: the damage suffered by the patient, the error of conduct on the part of the medical professional and the nexus, which consists of the relationship between the damage and the error.^10^

For all the themes exposed above about medical error, it was noted the need to explore the statistics on medical error in Brazil, especially in the area of Otorhinolaryngology, due to the lack of these studies.

Otorhinolaryngology is a specialty with a wide range of activities and, due to its extension, includes several “sub-specialties” such as Rhinology, which treats affections of the nose and paranasal sinuses, Otology, which takes care of diseases of the inner, middle and outer ears; Pharyngology, which takes care of diseases of the pharynx, Laryngology, which takes care of the vocal folds and voice, Otoneurology, which deals with labyrinth and balance disorders, Facial Aesthetic Surgery, which treats aesthetic and functional deformities of the face, and there are also otorhinolaryngologists with areas of expertise in Sleep Medicine, which treats snoring and sleep apnea, and Phoniatry, which deals with language disorders.

In Brazil, there are few rulings that deal with the civil consequences of otorhinolaryngology.^11^

The Federal and Regional Councils of Medicine were created through Decree Law 7.955 on September 13, 1945 to supervise the practice of medicine and were transformed into autarchies by Federal Law 3268/1957, which also defined the penalties possibly applied by the Councils: Penalties A, B, C, D and E.

A. Confidential warning in a reserved notice.

B. Confidential censorship in a reserved notice.

C. Public censorship in official publication.

D. Suspension of professional practice for up to 30-days.

E. Revocation of professional practice.

Because most physicians in general and Otorhinolaryngologists are concentrated in the State of São Paulo,^8^ this study surveys the cases of complaints of medical errors against specialists in Otorhinolaryngology with Specialist Qualification Registration registered in CREMESP that were processed between the years 2010 and 2020, being an excellent sample, whose results can be extrapolated to the entire national territory.

## Methods

The project was approved by the Research Ethics Committee (CEP) of the Escola Paulista de Medicina on February 7, 2023, under Opinion number 65387122.1.0000.5505.

A survey was carried out of all Inquiries and Professional Ethical Processes initiated against physicians with Professional Qualification Registration (RQE) in the area of Otorhinolaryngology at CREMESP, between the years 2010 and 2020. The numbers of the referred Inquiries and Processes were extracted from the database and were examined (whether physical or digitized) by a single examiner (author) who is currently a Counselor, for the collection of the following data:

• Complaints of medical error and the types of error: malpractice, recklessness or negligence
• Outcome of the Investigation (Filing, Conciliation, Conduct Adjustment Term [TAC] or initiation of a Professional Ethical Process [PEP])
• Outcome of the Professional Ethical Process (Non-Culpability, Culpability)
• Types of Penalties Applied at the Time of Culpability (Penalties A, B, C, D or E, according to article 22 of Law 3.268/57).

Due to the content being extremely confidential, only the first author, a Counselor of the 2023‒2028 administration, had access to the records, as she is bound by procedural secrecy. The names of the parties, registration number with the Regional Council of Medicine of the doctors involved, the fact that occurred and any other data that may lead to the identification of the parties will not be disclosed, following the General Data Protection Law (LGPD).

The findings were divided according to the areas of otorhinolaryngology (Rhinology, Snoring and Apnea, Otology, Pharyngology, Laryngology, Otoneurology, General Otorhinolaryngology and Facial Aesthetic Surgery) and the types of medical errors attributed to the respondent (investigations)/accused (professional ethical processes): Malpractice, Recklessness or Negligence. The examiner read the inquiries and processes and, according to the initial complaint, classified them within one of the 3 modalities of medical errors and within the subspecialties of Otorhinolaryngology, if any, or in General Otorhinolaryngology.

## Results

A total of 98 files were raised, 81 of which were archived inquiries and 17 were Professional Ethics Processes.

Among the 81 inquiries involving medical error in one of the 3 modalities raised in the survey, 35 of them (43.2%) were complaints for negligence, 23 (28.3%) for malpractice and 23 (28.3%) for recklessness.

Regarding the areas within Otorhinolaryngology, the errors were distributed as follows:

Negligence: Of the 35 negligence files, 20 were related to Otology (57.1%), 9 to Pharyngology (25.71%), 4 to Rhinology (11.4%), 1 to Laryngology (2.8%) and 1 to General Otorhinolaryngology (1%).

Malpractice: Of the 23 malpractice files, 10 were related to Otology (43.4%), 9 to Rhinology (39.1%), 2 to Pharyngology (8.6%) and 2 to Laryngology (8.6%).

Recklessness: Of the 23 files, 13 were related to Otology (56.5%), 5 to Rhinology (21.7%), 4 to Pharyngology (17.3%) and 1 to Laryngology (4.3%) (Table 1, Table 2).

**Table 1:** Investigations involving medical error.

Areas of Otorhinolaryngology	Malpractice	Recklessness	Negligence
Rhinology	39.1%	21.7%	11.4%
Snoring and apnea	0%	0%	0%
Otology	43.4%	56.5%	57.1%
Pharyngology	8.6%	17.3%	25.7%
Laryngology	8.6%	4.3%	2.8%
Otoneurology	0%	0%	0%
General ENT	0%	0%	2.8%
Facial Aesthetics Surgery	0%	0%	0%

**Table 2:** Professional Ethics Processes involving medical error.

Areas of Otorhinolaryngology	Malpractice (23.5%)	Recklessness (35.2%)	Negligence (41.1%)
Rhinology	0%	33%	85.7%
Snoring and apnea	0%	0%	0%
Otology	0%	33%	0%
Pharyngology	25%	16.6%	14.2%
Laryngology	50%	16.6%	0%
Otoneurology	0%	0%	0%
General ENT	0%	0%	0%
Facial Aesthetics Surgery	25%	0%	0%

Among the 17 Professional Ethics Processes surveyed that involved medical error in Otorhinolaryngology, 7 of them were due to complaints of Negligence (41.1%), 6 due to Recklessness (35.2%) and 4 due to complaints of Malpractice (23.5%).

Regarding the areas of Otorhinolaryngology, they were distributed as follows:

Negligence: 6 cases related to Rhinology (85.7%), 1 case related to Pharyngology (14.2%).

Malpractice: 2 cases related to the area of Laryngology (50%), 1 case related to Pharyngology (25%) and 1 case related to Facial Aesthetics (25%).

Recklessness: 2 cases related to Rhinology (33%), 2 cases related to Otology (33%), 1 case related to Pharyngology (16.6%) and 1 case related to Laryngology (16.6%).

Of the total of 17 cases, 12 have already been definitively judged (final and unappealable) and 5 are in the investigation phase.

Of the 12 cases already judged, in 9 (75%) of them the accused doctor was found guilty and in 3 (25%) of them he was found not guilty.

Regarding the sentence, 3 (33.3%) of the 9 accused were sentenced to sentence A, 3 (33.3%) were sentenced to penalty B, 1 (11.1%) was sentenced to penalty C, 2 (22.2%) of the accused doctors were sentenced to sentence D, and none of the denounced doctors suffered the maximum penalty imposed by the Council, which is penalty E (Table 3).

**Table 3:** Penalties applied.

Penalties Applied	Percentage of culprits
Confidential Warning in Reserved Notice	33.3%
Confidential Censorship on Reserved Notice	33.3%
Public Censorship	11.1%
Suspension of Professional Practice for up to 30-days.	22.2%
Revocation of Professional Practice	0%

Regarding the articles charged, 5 (55.5%) of the 9 guilty were penalized for violating article 1° of the 2018 Code of Medical Ethics (CME), which says:

“It is forbidden for the doctor to cause harm to the patient, by action or omission, characterized as malpractice, recklessness or negligence”.

The others were guilty in various articles of the Code of Medical Ethics (in one or more articles), such as 2, 4, 17, 18, 19, 21, 22, 32, 34, 35, 37, 57 and 87.

The areas of otorhinolaryngology that accounted for the highest number of culprits were Rhinology and Pharyngology, with 33.3% each, followed by Otology, Laryngology and Facial Aesthetics, with 11.1% each (Table 4).

**Table 4:** Culpability by area of Otorhinolaryngology.

Areas of Otorhinolaryngology	Number of culprits
Rhinology	3 (33.3%)
Snoring and apnea	
Otology	1 (11.1%)
Pharyngology	3 (33.3%)
Laryngology	1 (11.1%)
Otoneurology	
General Otorhinolaryngology	
Facial Aesthetics	1 (11.1%)

## Discussion

The results above showed that Otorhinolaryngology is a specialty that is relatively little triggered by medical error in the ethical sphere, because in 10-years, 81 inquiries were opened, an average of 8-per year and only 17 lawsuits, that is, less than two lawsuits per year.

Among the 98 complaints involving medical errors in Otorhinolaryngology between 2010 and 2020, which gave rise to investigations to investigate these complaints, only 17 of them originated Professional Ethical Processes, which means that the complaints were analyzed by the Counselors in the Chambers of Investigations, and the vast majority were filed because they did not constitute evidence of ethical infractions.

The complaints whose assessment in the Inquiry phase pointed to the possible existence of ethical illegalities on the part of the professionals, gave rise to ethical processes for better investigation and right of defense of the accused doctors.

Of these 17 cases, 12 have already been judged and are final and unappealable, 4 are still in progress and one is in the process of appealing after the decision of the judgment.

With regard to the severity of the penalties imposed to the otorhinolaryngologists, in 66% of the cases non-public penalties were imposed, which are penalties A, the most lenient that the Council can apply.

For 33% of the culprits, public penalties were applied, which are penalties C and D, published in the official gazette after being final and unappealable judged.

There was no sanction of revocation of professional practice for any otorhinolaryngologist in the lawsuits raised between the years 2010 and 2020 whose complaints were about malpractice, recklessness and negligence.

In relation to the articles attributed to otorhinolaryngologists considered guilty, 55.5% of the authors were assigned Article 1º, which is applied when there is damage to the patient due to any of the 3 types of medical errors studied in this study: malpractice, recklessness or negligence. In order for this article to apply, the occurrence of the actual damage must be proven.

For guilty doctors, other articles related to other types of infractions may have been applied, in addition to Article 1º that characterizes medical error, since the conduct is assessed as a whole and a doctor who was, for example, negligent, may also have failed to prepare a legible medical record (Article 87 of the CME) or failed to use all available means and within his reach in favor of the patient (Article 32 of the CME), or attributing their failures to third parties (Article 6º of the CME), among other infractions and will be penalized for them as well, at the same time as being penalized for medical error, as the conduct is globally assessed during the professional ethical judgment.

Regarding the subspecialties within Otorhinolaryngology, Otology is the area that received the most complaints filed, followed by Rhinology and Pharyngology.

Within the complaints that had the materiality to proceed with an investigation in the procedural phase, Rhinology is in the first position, with 47% of the complaints, followed by Pharyngology and Laryngology, making up 17.6% of the total complaints each. Otology comes next, making up 11.7% of the cases and Facial Aesthetic Surgery with 5.8%.

## Conclusion

With the rampant increase in the opening of medical schools and the decline in the quality of education, these statistical data need to be monitored. Studies of this type are rare in Brazil and need to be encouraged in order to have a picture of the reality of medical complaints in the country.^12^

The detection of the main complaints opens up a range of possibilities for us to work on the prevention of the occurrence of medical error, which brings numerous benefits to public health and to the country's economy.

## ORCID ID

Regina Maria Marquezini Chammes: 0000-0002-7612-5382

Reginaldo Raimundo Fujita: 0000-0002-0222-3596

## Funding

Main author (Regina Maria Marquezini Chammes) own recurses.

## Declaration of competing interest

The authors declare no conflicts of interest.
